# BuqiTongluo Granule for Ischemic Stroke, Stable Angina Pectoris, Diabetic Peripheral Neuropathy with Qi Deficiency and Blood Stasis Syndrome: Rationale and Novel Basket Design

**DOI:** 10.3389/fphar.2021.764669

**Published:** 2021-10-18

**Authors:** Weidi Liu, Li Zhou, Luda Feng, Dandan Zhang, Chi Zhang, Ying Gao, behalf of the BOSS Group

**Affiliations:** ^1^ Dongzhimen Hospital, Beijing University of Chinese Medicine, Beijing, China; ^2^ Institute for Brain Disorders, Beijing University of Chinese Medicine, Beijing, China

**Keywords:** basket design, BuqiTongluo granule, efficacy, herbal medicine, randomized controlled trial

## Abstract

**Background:** BuqiTongluo (BQTL) granules are herbal phenotypic drugs for Qi deficiency and blood stasis (QDBS) syndrome. Its discovery relied primarily on knowledge of observable phenotypic changes associated with diseases. Although BQTL granules have been widely advocated by Chinese Medicine (CM) practitioners, its use lacks empirical support.

**Aim of the study:** In this basket trial, the efficacy of BQTL granules in multiple diseases that have the QDBS syndrome in common will be compared with placebo.

**Materials and Methods:** The BuqiTongluo granule for Qi deficiency and blood stasis syndrome (BOSS) study is a basket herbal trial (ClinicalTrials.gov, NCT04408261). It will be a double-blinded, randomized, placebo-controlled, parallel, multicenter, clinical trial. In total, 432 patients (1:1:1 ischemic stroke, stable angina pectoris, and diabetic peripheral neuropathy), who meet the operationalized diagnostic criteria for QDBS syndrome, have been recruited and randomized in a ratio of 1:1 to receive 6 weeks’ treatment with BQTL granules or placebo. The primary outcome is the change in the QDBS syndrome score at week 6 from baseline. Secondary outcomes include objective outcome measures for the three diseases and adverse events. Omics will help to understand these responses by molecular events.

**Conclusion:** QDBS syndrome is a common phenotypic marker that was hypothesized to predict whether patients with multiple diseases would respond to this targeted therapy. No previous basket trial has assessed the potential efficacy of an herbal intervention for multiple diseases. The unique promise of the trial is its ability to exploit a disease phenotype to discover novel treatments for three diseases for which the root cause is unknown, complex, or multifactorial, and for which scientific understanding is insufficient to provide valid molecular targets.

## Introduction

Implementation of innovative design strategies in the development of efficacious and safe herbal medicines is of major interest to patients, the pharmaceutical industry, and regulators. In China, the National Medical Products Administration (NMPA) has supported various joint efforts to develop new methodologies for increasing the efficiency of clinical trials in complicated diseases. These design strategies include integrated herbal medicine protocol designs, as well as another closely related emerging concept, the basket trial design (Yuan, 2021). The term “basket trial” refers to a design developed to enroll individuals with multiple diseases and one (or a combination of) drug targets in cohorts within a trial (Cunanan et al., 2017). Basket designs have been applied positively in oncology trials dealing with multiple types of cancer (Park et al., 2020).

In Chinese Medicine (CM) theory, multiple diseases in different people may be treated in the same manner (Zhai et al., 2020). Under this treatment principle, the same strategy is used to treat patients with multiple diseases who have the same syndrome (Zheng) (Jiang et al., 2014). Such syndromes provide a massive amount of information in terms of herbal products and the clinical symptoms for which they are used therapeutically, which are the observable disease phenotypes that are crucial for clinical diagnosis and treatment (Miao et al., 2012). The change in the syndrome reflects either remission or progression of the disease.

We have adopted the concept of innovative basket trial design beyond the field of cancer research and have now developed a Phase II herbal trial protocol for treatment of patients with ischemic stroke, stable angina pectoris, and diabetic peripheral neuropathy, who share the same syndrome target [Qi deficiency and blood stasis (QDBS) syndrome] for herbal therapy. QDBS syndrome is one of the basic CM syndromes that is most strongly related to various diseases, including vascular, cardiovascular, and cerebrovascular diseases, and guides the use of herbal medicine (Zhai et al., 2020). It is a common and core pathogenesis of multiple diseases (Li et al., 2007), with a cluster of symptoms, including fatigue, shortness of breath, reticence to speak, spontaneous sweating, pale or dark complexion, local stabbing pain, pale purple tongue, or pale dark tongue. QDBS syndrome is thought to be the basic pathogenesis of ischemic stroke (Fan et al., 2014), coronary artery disease (Hui et al., 2010), and diabetic peripheral neuropathy (Miao et al., 2003) according to CM. Previous studies have demonstrated that changes in the phenotype of QDBS syndrome in patients with ischemic stroke, coronary artery disease, and diabetic peripheral neuropathy were associated with improvement in both symptoms and clinical outcomes of these three diseases (Zhou and Wang, 2014). Herbal medicine adopts a broad pharmacological approach to treat complicated diseases, by deploying a combination of herbal medicines with different treatment effects.

BuqiTongluo (BQTL) granule is a typical herbal formulae created by Yong-yan Wang, a famous Chinese medical scientist, to tonify Qi and promote blood circulation (Xie, 2020). The herbal formulae originates from Yi Lin Gai Cuo (Correcting the Errors in the Forest of Medicine) (Wang, 2007). It is composed of eight herbs, including Hedysarum polybotrys Hand.-Mazz (Hongqi), Panax notoginseng (Burkill) F.H.Chen (Sanqi), Alisma plantago-aquatica subsp. orientale (Sam.) Sam (Zexie), Angelica sinensis (Oliv.) Diels (Danggui), Ligusticum chuanxiong Hort (Chuanxiong), Periostracum Cicadae (Chantui), Curcuma aromatica Salisb (Yujin), Neolitsea cassia (L.), Kosterm (Guizhi). Now CM physicians often apply BQTL herbal formulae to treat nerve damage, cardiovascular and cerebrovascular diseases in clinical practice.

Among the herbs cited above, Hedysarum polybotrys Hand.-Mazz. contributes to regulating Qi. According to the Chinese medicine compatibility theory of “sovereign, ministerial, adjuvant and messenger”, the Hedysarum polybotrys Hand.-Mazz is sovereign herb in the BQTL, and it also showed pharmacological activity in well designed experiments. Hedysarum polybotrys polysaccharide (HPS) has significant protective effect against heart and brain hypoxia (Dong et al., 2013). It can protect the endomembrane barrier (Wang et al., 2013), promote peripheral nerve regeneration and improve the recovery of nerve function (Wei et al., 2009), and enhance nerve amplification effect by encouraging proximal axons to grow more lateral buds and effectively improve peripheral nerve (Wang et al., 2013). Panax notoginseng saponins (PNS) is isolated from the roots and rhizomes of Panax notoginseng (Burkill) F.H.Chen, a highly valued Chinese materia medica with the efficacy of promoting blood circulation and removing blood stasis. PNS has been shown to exert strong anti-inflammatory effects against atherosclerosis-related cardiac-cerebral vascular disease (Wan et al., 2009). A new study also suggested that PNS exerted long-term neuroprotective effects that assisted in stroke recovery (Zhou et al., 2021). PNS ameliorates diabetic peripheral neuropathy by attenuating electrophysiological, circulatory and morphological alterations (Hao et al., 2017).

Taken together, BQTL herbal formulae has been widely used to treat multiple diseases involving the QDBS syndrome. However, no large, randomized trial has studied the effect of BQTL granules on QDBS syndrome. The aims of this basket randomized controlled trial (RCT) are firstly to optimize parameters and examine the feasibility of a subsequent phase III RCT through preliminary evidence on the clinical efficacy and safety of BQTL granules on three diseases (ischemic stroke, stable angina pectoris, and diabetic peripheral neuropathy) involving QDBS syndrome, as compared with placebo. Secondly, it aims to elucidate the mechanism by which BQTL granules exert an effect on QDBS syndrome.

## Materials and Methods

### Study Design

**FIGURE 1:**
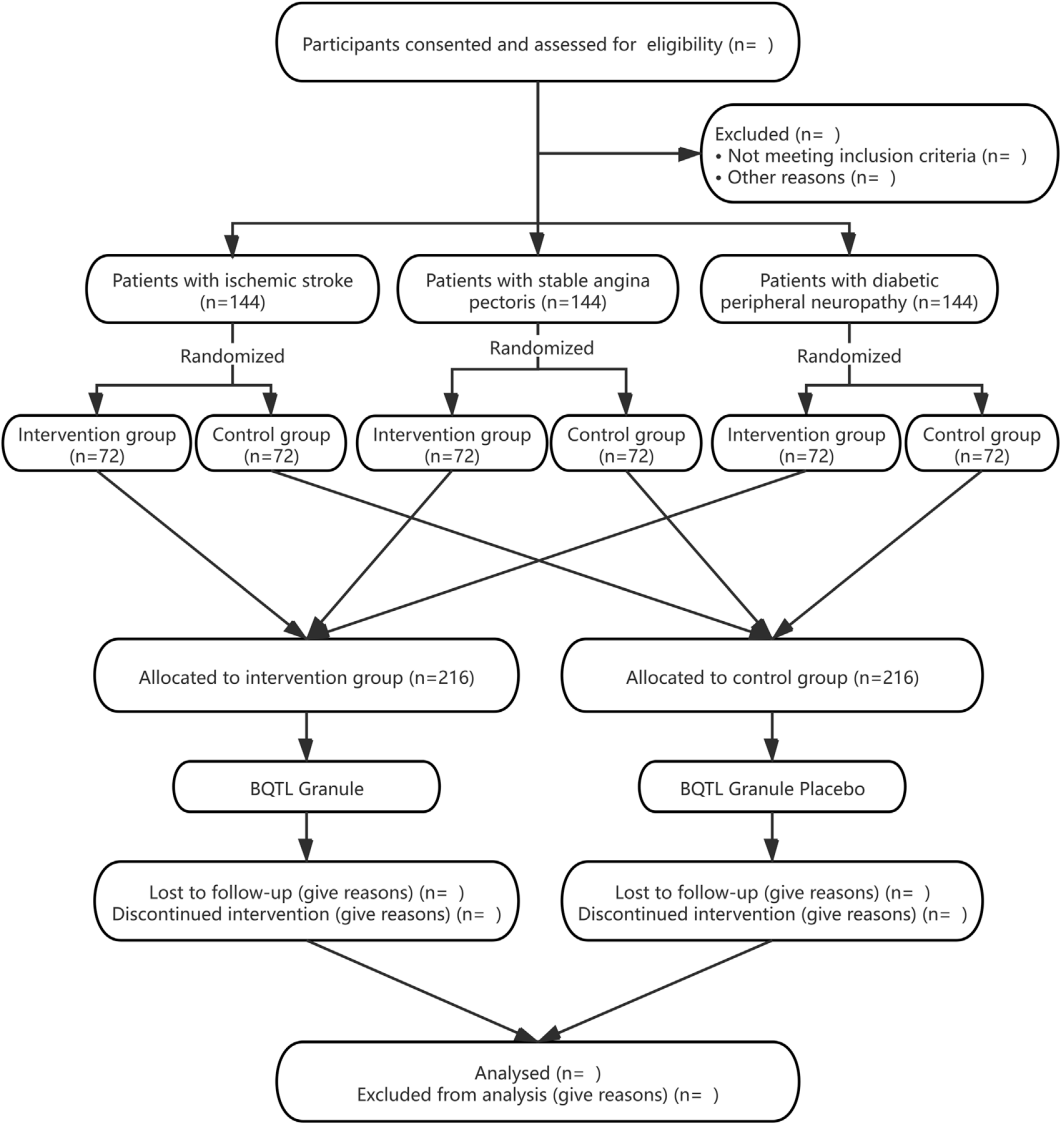
The flowchart of participants disposition throughout the study.

The BuqiTongluo granule for Qi deficiency and blood stasis syndrome (BOSS) study is a basket herbal trial studying the effect of BQTL granules across multiple cohorts of three diseases (ClinicalTrials.gov, NCT04408261). The BOSS protocol includes three multicenter randomized, double-blinded, placebo-controlled sub trials that will study BQTL granules in three independent patient populations: ischemic stroke (sub trial I), stable angina pectoris (sub trial II), and diabetic peripheral neuropathy (sub trial III). This study is currently open at 14 sites across China. In each sub trial, patients will be randomly divided into an intervention group (BQTL granule) and placebo group (placebo) using an 1:1 allocation ratio, adhering to the Standard Protocol Items: Recommendations for Interventional Trials (SPIRIT) statement (Chan et al., 2013). The study period includes 6 weeks of medication and 90 days of follow-up. The study procedure is summarized in the CONSORT diagram (Figure 1), and the schedule of enrolment, interventions, and assessments is summarized in Table1. The trial will be performed in accordance with the Declaration of Helsinki and Good Clinical Practice Guidelines. Informed consent will be obtained from all participants.

**TABLE 1:** The schedule of enrolment, interventions, and assessments in the BOSS trial.
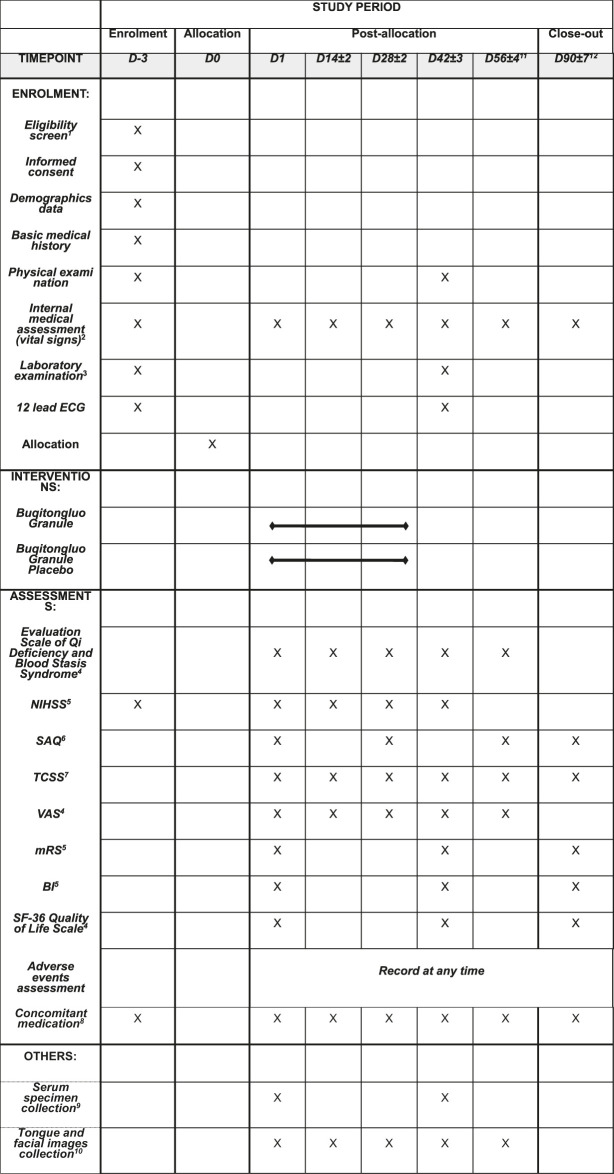

Note.

a Eligibility screen: Including inclusion and exclusion criteria, HbA1c, pregnancy test. Only patients with diabetic peripheral neuropathy will be tested for HbA1c, and only fertile women will be tested for blood pregnancy test within 24 h before the first medication.

b Vital signs: Including temperature, respiration, pulse and blood pressure.

c Laboratory examination: Including Routine Blood Examination (RBC, WBC, HGB, PLT, NEU, NE%) , Routine Urine Examination (GLU, LEU, PRO, KET, BLD, SG, pH) , Routine Stool Examination (RBC, WBC, fecal occult blood) , Blood Lipid Examination (TC, TG, LDL, HDL) , FBS, Electrolyte Examination (Na, K, Cl, Ca, P) , Liver Function (ALT, AST, GGT, ALP, TBIL) , Renal Function (BUN, Cr) , Coagulation Function (PT, APTT, TT, FIB, INR). Patients with diabetic peripheral neuropathy will also be tested for Urine Microalbumin and Urine NAG. During the trial, the investigator can decide whether to increase the safety indicator test items and times according to patients’ condition.

d Evaluation Scale of Qi Deficiency and Blood Stasis Syndrome, VAS and SF-36 Quality of Life Scale are applicable to all subjects.

e NIHSS, mRS and BI evaluation are only applicable to subjects with convalescence of ischemic stroke.

f SAQ evaluation is only applicable to subjects with stable angina pectoris of coronary artery disease.

g TCSS evaluation is only applicable to subjects with diabetic peripheral neuropathy.

h Concomitant medication: During the screening period and the whole process of the trial, antihypertensive agents are allowed to be used for blood pressure control. The concomitant medication will be recorded from 3 months before enrollment to the end of the trial.

i Serum specimen collection: The blood samples of 1/3 subjects (ie., the subjects with tongue and facial images collection) will be collected. The collection, processing and storage of blood samples will be carried out according to the “SOP for collection and management of clinical serum samples.”

j Tongue and facial images collection: All the subjects will use the mobile phone APP for tongue and facial images collection. And 1/3 subjects will use the TCM Tongue and Facial Imaging and Analysis Instrument for tongue and facial images collection.

k About D56: It refers to the 14th day after drug withdrawal.

l About D90: It refers to the 90th day after onset for subjects with convalescence of ischemic stroke, and refers to the 90th day after medication for subjects with stable angina pectoris of coronary artery disease and diabetic peripheral neuropathy.

### Participants

For inclusion, all participants will have a diagnosis of QDBS syndrome and should be aged from 35 to 80 years. QDBS syndrome follows the Guideline for clinical research of new Chinese medicine (The State Drug Administration, 2002).

For the ischemic stroke trial (sub Trial I), patients will be diagnosed with ischemic stroke (Sacco et al., 2013), with an interval from onset to recruitment of 14–30 days, and will have a National Institutes of Health Stroke Scale (NIHSS) score ≥4 and ≤22 (Kasner, 2006). Patients will be excluded from sub Trial I if they have a confirmed secondary stroke caused by tumors, brain trauma, or hematological diseases by clinical examination. Patients with other conditions that lead to motor dysfunction (e.g., lameness, osteoarthrosis, rheumatoid arthritis, gouty arthritis), which render a neurological function examination unlikely, will also be excluded.

In the stable angina pectoris trial (sub Trial II), patients will be included if they are diagnosed with stable angina pectoris and have a Canadian Cardiovascular Society classification of Angina Pectoris class I‒III (Knuuti et al., 2020). Patients will be excluded from this trial if they have had acute coronary syndrome or unstable angina pectoris in the previous 3 months, or have other heart diseases (e.g., cardiomyopathy, pericardial disease) such as severe cardiopulmonary insufficiency (congestive heart failure NYHA class IV, severe abnormal pulmonary function), or severe arrhythmias (e.g., rapid atrial fibrillation, atrial flutter, paroxysmal ventricular tachycardia).

In the diabetic peripheral neuropathy trial (sub Trial III), patients with a diagnosis of diabetic peripheral neuropathy will be included (Executive summary, 2014). Patients will be excluded if they have HbA1c >10% during the screening period, have had acute, critical diabetes mellitus conditions in the previous 3 months (e.g., hyperglycemia and hypertonic syndrome, diabetic lactic acidosis, diabetic ketoacidosis), or have severe heart disease, brain disease, or kidney disease. Moreover, patients with spinal cord injury, cervical or lumbar vertebral disease (nerve root compression, spinal stenosis, cervical or lumbar vertebra degenerative disease), or sequelae of cerebrovascular disease, neuromuscular junction, or muscular disease; or neuropathies caused by other diseases (e.g., Guillain-Barré syndrome, chronic inflammatory demyelinating polyneuropathy (CIDP), Vitamin B deficiency, hypothyroidism, alcoholism, or severe arteriovenous vasculopathy such as venous embolism, lymphangitis) will be excluded.

Additionally, patients with uncontrolled hypertension (systolic blood pressure ≥160 mmHg or diastolic blood pressure ≥100 mmHg), or renal or hepatic insufficiency (hepatic insufficiency defined as an alanine aminotransferase (ALT) or aspartate aminotransferase (AST) value that is 1.5 times the upper limit of normal; renal insufficiency defined as a serum creatinine concentration value that is above the upper limit of normal) will be excluded from all trials. Patients with other conditions or mental disorders that, according to the judgment of investigators, would restrict evaluation of mental function or render outcomes or follow-up unlikely to be assessable will also be excluded. Furthermore, pregnant or lactating women, or women who are planning a pregnancy within the next few years, patients who are allergic to the study drug or have a severely allergic constitution, those with a yellow, thick, slimy tongue coating, and those who have participated in other drug or device clinical trials in the past 3 months will also be excluded from all trials.

### Sample Size

For a main phase II trial assessing natural drugs, according to the NMPA recommendation (No. 28, 2007 and No. 109, 2018) and data from our pilot study, the sample size should not be less than 60 per treatment arm for each disease to estimate the parameter. We enrolled 432 participants to allow for dropouts.

### Recruitment

A total of 432 patients who fulfill the screening criteria will be recruited at 14 Good Clinical Practice (GCP)-approved hospitals in China. Local advertisements will be used for recruitment. A contract research organization will help to monitor the on-schedule recruitment progress and take measures in a timely manner. Investigators will provide research information, such as the purpose, the procedures, the potential risks and benefits of participants through standardized interviews before their participation. Written informed consent will then be obtained from all participants.

### Randomization, Allocation Concealment, and Blinding

All eligible patients who consent to participation will be randomized into either the BQTL granule group or the placebo group in an 1:1 ratio. A random sequence table will be generated by using Strategic Applications Software (SAS, version 9.4, SAS Institute, Inc., Cary, NC, United States), and randomization will be conducted using a central web-based interactive randomization service system (CIMS, Chengdu, China) with permuted blocks. The system automatically randomizes patients and generates the randomization code and drug code corresponding to the assigned treatment. The system will not release the randomization code until the patient has been recruited into the trial to ensure allocation concealment. Randomization will be conducted without any influence from research clinicians. All participants, physicians, nurses, data managers, statisticians, and other staff will be blinded to the treatment allocations until the trial is completed. In case of emergency, the principal investigator of each center, who has the only authority to view the blind codes, can log into the central randomization system for emergency unblinding. To ensure the implementation of blinding, the placebo will be identical to the BQTL granule in physical appearance, sensory perception, packaging, and labeling, and will have no pharmaceutical activity.

### Drug Administration

The BQTL granules contained Hedysarum polybotrys Hand.-Mazz (Hongqi), Panax notoginseng (Burkill) F.H.Chen (Sanqi), Alisma plantago-aquatica subsp. orientale (Sam.) Sam (Zexie), Angelica sinensis (Oliv.) Diels (Danggui), Ligusticum chuanxiong Hort (Chuanxiong), Periostracum Cicadae (Chantui), Curcuma aromatica Salisb (Yujin), and Neolitsea cassia (L.), Kosterm (Guizhi). The criteria for the quality of these ingredients were in accordance with the 2015 Chinese pharmacopoeia. Eligible patients will receive BQTL granules or placebo (10 g) (Table 2) dissolved in boiled water, administered orally three times a day for 6 weeks (one sachet per time). The study drug will be prepared by Shaanxi Buchang Pharmaceuticals Co., Ltd., China. The quality control of the production process strictly adhered to the good manufacturing practice (GMP) of national drug production.

**TABLE 2:** Standard formulation of BuqiTongluo Granule.

Pinyin name	Scientific name	Proportion (g)
Hongqi	Hedysarum polybotrys Hand.-Mazz	500
Sanqi	Panax notoginseng (Burkill) F.H.Chen	100
Zexie	Alisma plantago-aquatica subsp. orientale (Sam.) Sam	333
Danggui	Angelica sinensis (Oliv.) Diels	333
Chuanxiong	Ligusticum chuanxiong Hort	200
Chantui	Periostracum Cicadae	100
Yujin	Curcuma aromatica Salisb	200
Guizhi	Neolitsea cassia (L.) Kosterm	100

### Interventions

Eligible patients will be randomized in equal proportions between the BQTL granule groups and placebo groups, receiving either BQTL granules or placebo. The granules will be dispensed through the clinical trial pharmacies of the 14 hospitals, complying with the standard of pharmacy practice. Two fully registered CM practitioners will be responsible for clinical diagnosis and prescription. During the trial, it will be forbidden to use acupuncture, CM decoctions (compound granules), Chinese medicine injections, Chinese patent medicines (including external use), and external washing with traditional Chinese medicine and health products with a composition or efficacy similar to the study drug. Participants will be allowed to accept standard rehabilitation treatment and use necessary drugs that do not affect the evaluation of study parameters for concomitant diseases. The use of drugs should be standardized according to the guidelines, and the name, dosage, times, and time of use must be recorded for analysis and report.

### Primary Outcomes

The primary outcome of the study is improvement in QDBS syndrome, defined as a change in the national approved QDBS syndrome evaluation scale from before to after the 6-week treatment. The QDBS syndrome evaluation scale (Wang et al., 2015) was chosen because of its practical use in the evaluation of the QDBS syndrome. The scale is a 21-item clinician-rated scale with anchored item descriptions. It lists 21 clinical symptoms and physical signs related to the QDBS syndrome. The scale ranges from 0 to 51 with 51 indicating the worst score possible. The higher the score, the more severe the degree of the syndrome. The scale has been developed, validated, and applied in the Department of Neurology, Cardiology, Endocrinology, and multiple investigators have used this method to assess patients with QDBS syndrome. This scale has excellent inter-rater reliability and internal consistency. It is NMPA’s recommended outcome measure in Guideline for clinical research of new Chinese medicine drugs with syndrome.

### Secondary Outcomes

For sub Trial I, secondary outcomes will be the following: Neurological impairment will be evaluated using the NIHSS (Time Frame: baseline, and on days 14, 28, and 42 during treatment). Self-rating symptoms will be evaluated using a visual analog scale (VAS) (Bijur et al., 2001). This will include VAS scores for limb numbness, swelling of hands or feet, and spontaneous sweating (hemilateral sweating) (Time Frame: baseline, and on days 14, 28, and 42 during treatment, as well as at day 14 after treatment). Continuous changes in the Modified Rankin Scale score (Uyttenboogaart et al., 2005) (Time Frame: baseline, at day 42 during treatment, and at day 90 after onset) will also be recorded. Activities of daily living will be measured using the Barthel Index (BI) score (Uyttenboogaart et al., 2005) (Time Frame: baseline, at day 42 during treatment, and at day 90 after onset).

For sub Trial II, secondary outcomes will include the following: Changes in the Seattle Angina Questionnaire (SAQ) score (Time Frame: baseline, at day 28 during treatment, at day 14 after treatment, and at day 90 after recruitment). Self-rating symptoms will be evaluated using a VAS for chest tightness, chest pain, palpitation, fatigue, and spontaneous sweating (Time Frame: baseline, and on days 14, 28, and 42 during treatment, as well as at day 14 after treatment).

In sub Trial III, the secondary outcomes will be as follows: Changes in the Toronto Clinical Scoring System (Bril and Perkins, 2002) (Time Frame: baseline, and on days 14, 28, and 42 during treatment, day 14 after treatment, as well as day 90 after recruitment). Self-rating symptoms will be evaluated using a VAS for local pain, limb numbness, and paresthesia (e.g., burning sensation, formication, electrical sensation) (Time Frame: baseline, and on days 14, 28, and 42 during treatment, as well as at day 14 after treatment).

For all sub trials, quality of life will be measured as a secondary outcome, using the 36-Item Short Form Survey (SF-36) (Time Frame: Baseline, at day 42 during treatment, and at day 90 after onset/recruitment). Blood, urine, and feces samples will be collected at baseline and day 42 for metabolomics, proteomics, microbiota, and flow-cytometry studies.

### Safety Outcomes

The safety outcomes will include any adverse events and clinically meaningful changes in vital signs or laboratory parameters during the trial. Participants will be asked to report any abnormal reactions occurring during the trial to the investigators. At week 6, participants will undergo liver function and renal function tests to monitor hepatotoxicity and nephrotoxicity.

### Adverse Events Management

All details of related and unexpected adverse events, including time of occurrence, degree and duration, suspected causes, and effective measures and outcomes will be recorded. The investigators will immediately take appropriate treatment measures for the participants, report and follow-up, and assess the relatedness of the event to the study drug from a clinical point of view. Any adverse event will be treated suitably and recorded accurately and completely. The causality between adverse events and intervention will be assessed by a causality assessment tool, the World Health Organization-Uppsala Monitoring Centre (WHO-UMC) case causality assessment, to evaluate the likelihood that an adverse event is associated with the intervention. The causality assessment will be accomplished by a combined assessment taking into account the clinical-pharmacological aspects of the case history and the quality of the documentation of the observation with a table of predefined statements and classifies events as the following categorization: certain, probable/likely, possible, unlikely, and two sub-categories: conditional/unclassified (when information pending), and unassessable/unclassifiable (when sufficient information is not available).

### Data Collection and Management

Data will be collected at baseline, at 14, 28, 42, and 56 days post-allocation, and at the 90 days follow-up. To promote data quality, each site’s investigator will be trained centrally regarding the study requirements, including standardized evaluation of scales involved in the trial, operation of the CM tongue and facial imaging and analysis instrument, requirements for serum specimen collection, and on eliciting information from participants in a uniform and reproducible manner.

An electronic data capture system will be used in this study. The investigator/clinical research coordinator (CRC) will input the original data into the electronic data capture system accurately, in a timely, complete, and standard manner. After data entry, data may not be changed at will. If data entries need to be modified, the investigator/CRC will need to record the reason for modification according to the system prompt. All the operations in the system are traceable.

The data manager will develop a detailed data verification plan according to the protocol and case report form, including logic verification, scope verification, time window verification, consistency verification, and compliance verification. The efficiency indicators and key safety indicators should be fully verified to ensure the accuracy and integrity of the data. Data verification should be carried out in the case of an unknown test group, and the generated data query content should avoid deviation or induced questions.

After all the data are inputted and queried, a blind review meeting will be held to determine the data set division. After reaching a consensus on all data questions, the sponsor, principal investigator, data manager, and statistician will jointly sign the approval document for locking the database. After obtaining approval, the data manager will execute the database locking operation and remove the system operation authority of the relevant personnel. The study documents will be retained in a secure location for at least 5  years after trial completion.

### Quality Control and Data Monitoring

The trial is managed by the Dongzhimen Hospital, Beijing University of Chinese Medicine. The protocol compliance, safety, and the trial data will be supervised by the Data and Safety Monitoring Board (DSMB), an independent group of experts that advises funding agencies and study investigators. DSMB members include experts from different fields (Western Medical Sciences, Chinese Medicine, Clinical Epidemiology, and Statistics). An auditing will be conducted twice a month during the enrollment period and every month during the follow-up period, and the process will be independent from investigators.

### Statistical Methods

Statistical analysis will be performed using Statistical Analysis System version 9.4 (SAS Institute Inc., Cary, North Carolina, United States) statistical software packages. Outcome measurements will be analyzed using full analysis sets and per protocol sets according to intention-to-treat (ITT) analysis. Safety analysis will be performed in a safety set, which is defined as a subset of subjects who received at least one treatment and had actual safety indicators record data. The statistical analysis will include baseline characteristics of participants, compliance and concomitant medication, efficacy analysis and safety analysis. For continuous data, we will describe the results using the mean (standard deviation, SD), maximum, minimum, median and non-parametric test median (quartile deviation, QD). Categorical data will be described as absolute values and proportions. For continuous outcomes, paired *t*-test or Wilcoxon signed-rank test will be used to analyze significant differences between baseline and each time point. The Wilcoxon rank-sum test will be employed for comparisons between treatment groups. Chi-squared test will be used for categorical data and Wilcoxon rank-sum test will be used for ranked data. Analysis of covariance (ANCOVA) will be used to control potential confounding variables.

We will use a two-sided 5% significance level and 85% power. Baseline characteristics in each group will be analyzed using descriptive statistics. Compliance analysis will be based on full analysis sets, and analysis of concomitant medications will be based on safety sets. Regarding the primary outcome variable, between-group comparisons of the change in syndrome score will be analyzed between pre- and post-treatment using paired t-test or Wilcoxon signed-rank test. As to the secondary outcomes, comparisons of the changes in NIHSS score in sub Trial I, the Seattle Angina Questionnaire score in sub Trial II, and the Toronto Clinical Scoring System in sub Trial III, will be analyzed using paired t-test or Wilcoxon signed-rank test between baseline and each time point. The Wilcoxon rank-sum test will be employed for comparisons between treatment groups. Any factors impacting efficacy, such as age and sex, should be taken into account as covariate. Safety will be analyzed in terms of all the adverse events occurred during the trial, and the incidence will be compared between groups using the chi-squared test or Fisher’s exact probability method.

Last observation carry forward (LOCF) approach will be used to impute missing data of primary outcome according to intention-to-treat analysis. A sensitivity analysis will be conducted to determine the robustness of the results under the missing at random assumption, to evaluate the role of lost to follow-up (i.e., participants who did not follow-up to V4), by using the LOCF method. And a *p* value of less than 0.05 will be considered statistically significant.

## Discussion

The BOSS trial is the first study that implements basket design in the context of herbal medicine (Yuan, 2021). Before BOSS, basket design is conceptual phase in this field. Basket trials have been developed as an efficient way to screen for experimental therapeutics across multiple patient populations in the early phase of drug development. Rather than being viewed as opposing alternatives in herbal drug development, the basket trial design (different diseases treated with the same therapy) and conventional RCT design (one disease treated with one therapy) should be seen as complementary methodologies that can increase the odds of developing herbal drugs with promising phenotypic predictors. To our knowledge, the basket trial approach has not been fruitfully applied to evaluate the efficacy of herbal medicine for CM syndromes in patients with different diseases. BQTL granules are the first new CM drug approved for application in Phase II trials by the NMPA. A positive outcome of the BOSS trial would set the stage for future investigations using the basket trial design to increase the feasibility of herbal medicine for individuals with specific CM syndromes.

We present this protocol and a detailed statistical analysis plan prior to the analyses of any data in the BOSS trial. Recruitment for the study started on July 22, 2020, and the trial is expected to be completed in December 2022. The strengths of our trial include the high methodological standards of a double-blind, placebo-controlled, randomized clinical trial, and high external validity, with 14 sites, which will ensure robust results and reduces the influence of confounding covariates. We will report patient-centered outcomes and disease-related objective assessments of severity, measured using VASs. The trial will be monitored according to GCP standards. The proposed study may provide direct and convincing evidence to support BQTL granules as a treatment for improving QDBS syndrome, which could then be introduced into clinical settings. It will support symptomatic treatment of QDBS syndrome with BQTL granules, and holds potential to make a meaningful difference to patients. The unique promise of the BOSS trial is its ability to exploit a disease phenotype to discover novel treatments for three diseases for which the root cause is unknown, complex, or multifactorial, and for which scientific understanding is insufficient to provide valid molecular targets.

## Abbreviations

ALT, alanine aminotransferase; AR, Alisma plantago-aquatica subsp. orientale (Sam.) Sam.; AS, Angelica sinensis (Oliv.) Diels; AST, aspartate aminotransferase; BI, barthel index; BQTL, BuqiTongluo; CI, confidence interval; CIMS, clinical information management suite; CM, Chinese medicine; CIDP, chronic inflammatory demyelinating polyneuropathy; CRC, clinical research coordinator; DSMB, data and safety monitoring board; HPS, hedysarum polybotrys polysaccharide; GCP, good clinical practice; GMP, good manufacturing practice; LC, Ligusticum chuanxiong Hort.; NIHSS, national institutes of health stroke scale; NMPA, national medical products administration; NYHA, New York Heart Association; PNS, panax notoginseng saponins; QDBS, Qi deficiency and blood stasis; RCT, randomized controlled trial; SAQ, Seattle Angina Questionnaire; SAS, strategic applications software; SF-36, 36-item short form survey; VAS, visual analog scale.
