# Supplementary material for: BuqiTongluo Granule for Ischemic Stroke, Stable Angina Pectoris, Diabetic Peripheral Neuropathy with Qi Deficiency and Blood Stasis Syndrome: Rationale and Novel Basket Design
Source: Front Pharmacol. 2021 Oct 18;12:764669. doi: 10.3389/fphar.2021.764669 (PMC8558407; doi:10.3389/fphar.2021.764669)
Supplement: Supplementary file 1 [file DataSheet1.docx]

***Supplementary Files***

**Appendix Table 1 The identified constituents in BuqiTongluo Granules methanol extract by UPLC-Q-TOF-MS^E^.**

**Appendix Table 2  Standardized Protocol Items for BOSS.**

# Appendix Table 1. The identified constituents in BuqiTongluo Granules methanol extract by UPLC-Q-TOF-MS^E^^[[1]](#endnote-0)^.

| **Chromatographic peak** | **t_R_/min** | **Molecular formula** | **Measured value of quasi-molecular ion peaks（m/z）** | **Mass number error（mDa）** | **Adduct ion** | **Fragment ion** | **Chemical compound** | **assignment** |
| --- | --- | --- | --- | --- | --- | --- | --- | --- |
| 1 | 1.40 | C_8_H_8_O_4_ | 191.031 5 | 0.1 | +Na |  | 4-Hydroxy-3-methoxybenzoic acid | Danggui |
| 2 | 1.48 | C_5_H_9_NO_2_ | 116.031 5 | -7.2 | +H |  | L(-)-Proline | Chantui |
| 3 | 1.49 | C_3_H_7_NO_2_ | 112.043 1 | 6.2 | +Na |  | L-Alanine | Chantui |
| 4 | 1.51 | C_16_H_16_O_4_ | 273.112 4 | 0.3 | +H | 258.1285,242.0967, 238.0640,224.0486 | 2',7-Dihydroxy-4'-Methoxyisoflavan | Danggui |
| 5 | 1.52 | C_4_H_9_NO_2_ | 126.047 6 | -5.0 | +Na |  | DL-2-Aminobutyric Acid | Chantui |
| 6 | 1.53 | C_12_H_14_O_2_ | 213.087 7 | -0.9 | +Na | 177.1327,177.0921 174.0679,167.1113 | Z-ligustilide | Danggui |
| 7 | 1.54 | C_4_H_7_NO_4_ | 134.037 5 | -7.3 | +H |  | Aspartic acid | Chantui |
| 8 | 1.97 | C_4_H_6_O_4_ | 119.041 8 | 7.9 | +H |  | Succinic Acid | Danggui |
| 9 | 2.01 | C_10_H_10_O_3_ | 179.074 1 | 3.9 | +H | 165.0479,163.0801, 163.0320,149.0639 | 2-Methoxycinnamic acid | Guizhi |
| 10 | 2.31 | C_12_H_18_O_2_ | 217.123 0 | 3.1 | +Na | 179.1473 | Neocnidilide | Chuanxiong |
| 11 | 2.47 | C_15_H_24_O_4_S | 323.129 4 | 0.7 | +Na | 256.0862,236.0942, 210.0805 | Sulfoorientalol C | Zexie |
| 12 | 2.74 | C_15_H_26_O_4_S | 325.144 9 | 0.4 | +Na | 273.1563,247.1004, 235.1378,221.1219 | Sulfoorientalol A | Zexie |
| 13 | 3.67 | C_9_H_11_NO_3_ | 182.085 0 | 3.9 | +H | 164.0625,146.0530 | L-Tyrosine | Chantui |
| 14 | 4.00 | C_15_H_26_O_5_S | 319.158 0 | 0.6 | +H | 266.1342,253.1130, 245.0513,231.1065 | Sulfoorientalol B | Zexie |
| 15 | 4.84 | C_20_H_18_O_5_ | 361.101 6 | -3.0 | +Na | 323.0899,189.0471 | Demethoxycurcumin | Yujin |
| 16 | 5.14 | C_15_H_12_O_4_ | 257.076 5 | -4.3 | +H |  | Isoliquiritigenin | Hongqi |
| 17 | 6.00 | C_15_H_26_O_5_S | 319.158 0 | 0.6 | +H | 273.1871,229.0872, 219.1063,191.1068 | Sulfoorientalol D | Zexie |
| 18 | 6.39 | C_10_H_18_O_4_ | 225.111 9 | 2.1 | +Na | 187.1399,185.1232 | Sebacic acid | Danggui |
| 19 | 6.78 | C_31_H_44_O_8_ | 567.291 6 | -1.2 | +Na | 495.2352,466.2338, 397.2063,384.1889 | 23-Acetyl alismatolide | Zexie |
| 20 | 7.05 | C_15_H_12_O_4_ | 257.086 0 | 5.2 | +H | 243.1012,165.0459 | Liquiritigenin | Hongqi |
| 21 | 7.06 | C_54_H_92_O_23_ | 1 131.589 4 | -2.8 | +Na | 1052.5454,1026.5058, 994.5060,985.4772 | Ginsenoside Rb1 | Sanqi |
| 22 | 7.08 | C_9_H_8_O | 133.057 4 | -7.4 | +H |  | Cinnamaldehyde | Guizhi |
| 23 | 7.36 | C_12_H_18_O_2_ | 217.123 0 | 3.1 | +Na | 169.1257 | Cnidiumlactone | Danggui |
| 24 | 8.53 | C_42_H_72_O_14_ | 801.506 3 | 6.8 | +H | 783.4959,765.4867, 703.3948,690.3725 | Ginsenoside Rg1 | Sanqi |
| 25 | 9.03 | C_13_H_16_O_5_ | 275.088 0 | -1.0 | +Na | 221.0348,182.0900, 180.0730,168.0735 | Methyl 3,4,5-Trimethoxycinnamate | Hongqi |
| 26 | 9.45 | C_22_H_22_O_9_ | 431.131 0 | -2.6 | +H | 417.1509,415.1299, 413.1183,385.1372 | Ononin | Hongqi |
| 27 | 9.61 | C_19_H_16_O_4_ | 309.112 5 | 0.4 | +H | 293.1170,191.0784 189.0629 | Bisdemethoxycurcumin | Yujin |
| 28 | 9.74 | C_4_H_8_N_2_O_3_ | 155.039 9 | -2.9 | +Na |  | L-Asparagine | Chantui |
| 29 | 10.62 | C_12_H_16_O_2_ | 215.100 3 | -3.9 | +Na | 179.0998,175.1086, 165.0841,163.1046 | Senkyunolide | Chuanxiong |
| 30 | 10.70 | C_48_H_82_O_19_ | 963.554 9 | 2.6 | +H | 917.4650,912.5015, 881.4546,859.4376 | 20-O-Glucoginsenoside Rf | Sanqi |
| 31 | 10.95 | C_41_H_70_O_13_ | 793.471 1 | 0.2 | +Na | 753.4864,621.4370, 603.4252,493.3037 | Notoginsenoside R2 | Sanqi |
| 32 | 11.16 | C_21_H_20_O_6_ | 369.140 9 | 7.7 | +H | 353.0964,351.1185, 350.1093,335.0863 | Curcumin | Yujin |
| 33 | 11.52 | C_48_H_82_O_18_ | 947.555 4 | -2.0 | +H | 929.5565,818.4332, 795.4507,325.1074 | Ginsenoside Re | Sanqi |
| 34 | 12.18 | C_15_H_24_O_2_ | 237.178 7 | -6.2 | +H | 207.1322,179.0998, 160.0447 | Germacr-1(10)-ene-5,8-dione | Yujin |
| 35 | 12.32 | C_15_H_22_O_2_ | 235.163 5 | -5.8 | +H | 217.1520,210.1201, 191.1001,189.0845 | Isocurcumenol | Yujin |
| 36 | 12.33 | C_64_H_108_O_31_ | 1 373.694 3 | -0.5 | +H | 1355.6821,1323.6900, 1314.6417,295.0973 | Notoginsenoside T | Sanqi |
| 37 | 12.81 | C_63_H_106_O_30_ | 1 343.684 8 | 0.6 | +H | 1263.6189,753.4837, 423.3578,231.0437 | Notoginsenoside S | Sanqi |
| 38 | 12.82 | C_36_H_62_O_9_ | 639.446 6 | 0.0 | +H | 621.4365,605.4411, 423.3578,217.1883 | 20(S)-Ginsenoside Rh1 | Sanqi |
| 39 | 12.83 | C_36_H_60_O_8_ | 621.436 7 | 0.6 | +H | 605.4411,591.4249, 423.3578,217.1883 | Ginsenoside Rh4 | Sanqi |
| 40 | 13.22 | C_16_H_12_O_5_ | 285.070 5 | -5.3 | +H | 269.0735,267.0594, 255.0580,252.0362 | 7-hydroxy-3-(3-hydroxy-4-methoxyphenyl)-4H-chromen-4-one | Hongqi |
| 41 | 13.25 | C_59_H_100_O_27_ | 1 241.652 6 | 0.1 | +H | 605.4411,591.4249, 423.3578,217.1883 | Notoginsenoside Fa | Sanqi |
| 42 | 13.44 | C_12_H_8_O_4_ | 217.043 5 | -6.0 | +H | 202.0196,185.0163, 174.0243,151.0310 | 5-Methoxy psoralen | Danggui |
| 43 | 13.45 | C_15_H_22_O_2_ | 235.163 2 | -6.0 | +H | 205.1158,189.1200, 75.0685,161.0888 | Curcumenol | Yujin |
| 44 | 13.81 | C_58_H_98_O_26_ | 1 233.621 4 | -2.4 | +Na | .197.6357,934.4777, 877.5015,809.4329 | Notoginsenoside Fc | Sanqi |
| 45 | 13.84 | C_32_H_46_O_6_ | 549.318 5 | -0.2 | +Na | 499.3029,457.2925, 451.3170,445.2551 | 23-O-Acetylalisol J | Zexie |
| 46 | 13.95 | C_53_H_90_O_22_ | 1 079.600 8 | 1.2 | +H | 1049.5530,1010.5855, 767.5014,425.3742 | Ginsenoside Rc | Sanqi |
| 47 | 14.57 | C_16_H_12_O_4_ | 269.075 7 | -5.1 | +H, +Na | 255.0964,254.0512, 253.0897,253.0441 | Formononetin | Hongqi |
| 48 | 14.78 | C_9_H_6_O_2_ | 147.037 1 | -6.9 | +H |  | Coumarin | Guizhi |
| 49 | 14.81 | C_30_H_46_O_4_ | 493.329 7 | 0.8 | +Na | 441.3325,429.2971, 411.2878,393.2753 | Neoalisol A | Zexie |
| 50 | 14.92 | C_9_H_8_O_2_ | 149.052 8 | -6.9 | +H |  | Coumalic acid | Guizhi |
| 51 | 15.37 | C_31_H_52_O_5_ | 527.371 1 | 0.4 | +Na | 459.3798,441.3692, 423.3582,401.2664 | 25-methoxyalisol A | Zexie |
| 52 | 15.89 | C_25_H_40_O_6_ | 459.271 9 | 0.2 | +Na | 409.2496,404.2943, 387.2510,191.0999 | Oriediterpenoside | Zexie |
| 53 | 17.55 | C_47_H_80_O_17_ | 917.547 6 | 0.7 | +H | 749.4130,681.3899, 663.4163,511.3385 | Notoginsenoside Fe | Sanqi |
| 54 | 18.47 | C_47_H_80_O_18_ | 933.545 0 | 3.2 | +H | 625.3224,585.3324, 575.2656,574.2591 | Notoginsenoside R1 | Sanqi |
| 55 | 18.64 | C_30_H_46_O_5_ | 509.324 4 | 0.6 | +Na | 471.3430,453.3322, 249.1478,189.0843 | Alisol C | Zexie |
| 56 | 19.04 | C_30_H_48_O_5_ | 511.339 4 | 0.0 | +Na, +H | 471.3435,439.3531, 339.2635,187.1414 | Alisol F | Zexie |
| 57 | 19.11 | C_30_H_50_O_5_ | 513.354 7 | -0.3 | +Na | 441.3676,424.3013, 399.2842,371.2537 | 11-Deoxy-13β,17β-epoxy-alisol A | Zexie |
| 58 | 20.49 | C_32_H_50_O_6_ | 553.348 4 | -1.6 | +Na | 514.3585,495.3428, 318.2948,191.0998 | Alismaketone A 23-acetate | Zexie |
| 59 | 20.73 | C_30_H_50_O_6_ | 529.350 2 | 0.2 | +Na | 395.2532,231.1317 | 13,17-Epoxyalisol A | Zexie |
| 60 | 20.80 | C_30_H_48_O_6_ | 527.334 5 | 0.2 | +Na | 423.2700,393.2499, 369.2359,207.0951 | 16-Oxo- alisol A | Zexie |
| 61 | 21.25 | C_32_H_52_O_6_ | 555.365 4 | -0.2 | +Na | 515.3716,497.3603, 489.3539,479.3485 | Alisol E 23-acetate | Zexie |
| 62 | 21.25 | C_32_H_50_O_5_ | 515.371 9 | -1.2 | +H | 497.3603,489.3539 479.3485,453.3324 | 25-Anhydro-alisol A 24-acetate | Zexie |
| 63 | 21.58 | C_30_H_50_O_5_ | 513.355 1 | 0.1 | +Na | 443.3836,425.3730, 383.2522,339.2637 | Alisol A | Zexie |
| 64 | 22.77 | C_32_H_50_O_5_ | 515.371 8 | -1.3 | +H | 497.3550,485.3226, 467.3120,457.3272 | 25-Anhydro-alisol A 11-acetate | Zexie |
| 65 | 21.80 | C_29_H_50_O | 437.371 7 | -3.7 | +Na | 261.2152,243.2041 | Beta-Sitosterol | Hongqi |
| 66 | 23.79 | C_32_H_50_O_6_ | 531.367 0 | -1.0 | +H | 516.3794,502.3590 489.3522,446.3368 | Alisol F 24-acetate | Zexie |
| 67 | 23.82 | C_32_H_50_O_4_ | 521.360 9 | 0.7 | +Na | 484.3510,465.3360, 423.3587,415.2895 | 11-Deoxy-alisol B 23-acetate | Zexie |
| 68 | 24.47 | C_32_H_46_O_5_ | 511.341 1 | -0.7 | +H | 493.3265,469.3290, 451.3167,435.3217 | Alisol L 23-acetate | Zexie |
| 69 | 24.55 | C_12_H_14_O_2_ | 191.100 3 | -6.4 | +H | 177.0844,173.0891, 163.1046,161.0527 | E-Ligustilide | Danggui |
| 70 | 25.73 | C_30_H_48_O_4_ | 495.344 4 | 0.0 | +Na | 397.2696,381.2741, 377.2782,337.2479 | Alisol G | Zexie |
| 71 | 27.05 | C_30_H_48_O_3_ | 479.349 2 | -0.4 | +Na | 441.3681,423.3576, 411.2836,201.1571 | Ursolic acid | Danggui |
| 72 | 27.84 | C_32_H_50_O_6_ | 553.348 5 | -1.5 | +Na | 495.3420,455.3473, 339.2636,201.1570 | Alisol D | Zexie |

# Appendix Table 2 Standardized Protocol Items for BOSS.

| **Section/item** | **ltem No.** | **Description** | **Addressed on page number** |
| --- | --- | --- | --- |
| **Administrative information** | | | |
| Title | 1 | BuqiTongluo Granule for Ischemic Stroke, Stable Angina Pectoris, Diabetic Peripheral Neuropathy with Qi Deficiency and Blood Stasis Syndrome (BOSS): Rationale and Novel Basket Design | Page 1, line 1 |
| Trial registration | 2a | ClinicalTrials.gov: NCT04408261 | Page 4, line 38 |
| Protocol version | 3 | 13 Dec 2019 | Page 19, line 416 |
| Funding | 4 | This research was financially supported by the Beijing University of Chinese Medicine Project [2020-tsxk-001], the Chinese Medicine Inheritance and Innovation Talent Project-Leading Talent Support Program of National Traditional Chinese Medicine [2018, 12], the Dongzhimen Hospital Project [2020TSRC-002]. | Page 19, line 410 |
| **Introduction** | | | |
| Background and rationale | 6a | Implementation of innovative design strategies in the development of efficacious and safe herbal medicines is of major interest to patients, the pharmaceutical industry, and regulators. In China, the National Medical Products Administration (NMPA) has supported various joint efforts to develop new methodologies for increasing the efficiency of clinical trials in complicated diseases. These design strategies include integrated herbal medicine protocol designs, as well as another closely related emerging concept, the basket trial design. The term “basket trial” refers to a design developed to enroll individuals with multiple diseases and one (or a combination of) drug targets in cohorts within a trial. Basket designs have been applied positively in oncology trials dealing with multiple types of cancer. In Chinese Medicine (CM) theory, multiple diseases in different people may be treated in the same manner. Under this treatment principle, the same strategy is used to treat patients with multiple diseases who have the same syndrome. Such syndromes provide a massive amount of information in terms of herbal products and the clinical symptoms for which they are used therapeutically, which are the observable disease phenotypes that are crucial for clinical diagnosis and treatment. The change in the syndrome reflects either remission or progression of the disease.  We have adopted the concept of innovative basket trial design beyond the field of cancer research and have now developed a Phase II herbal trial protocol for treatment of patients with ischemic stroke, stable angina pectoris, and diabetic peripheral neuropathy, who share the same syndrome target (Qi deficiency and blood stasis [QDBS] syndrome) for herbal therapy. QDBS syndrome is one of the basic CM syndromes that is most strongly related to various diseases, including vascular, cardiovascular, and cerebrovascular diseases, and guides the use of herbal medicine. It is a common and core pathogenesis of multiple diseases, with a cluster of symptoms, including fatigue, shortness of breath, reticence to speak, spontaneous sweating, pale or dark complexion, local stabbing pain, pale purple tongue, or pale dark tongue. QDBS syndrome is thought to be the basic pathogenesis of ischemic stroke, coronary artery disease, and diabetic peripheral neuropathy according to CM. Previous studies have demonstrated that changes in the phenotype of QBDS syndrome in patients with ischemic stroke, coronary artery disease, and diabetic peripheral neuropathy were associated with improvement in both symptoms and clinical outcomes of these three diseases. Herbal medicine adopts a broad pharmacological approach to treat complicated diseases, by deploying a combination of herbal medicines with different treatment effects. | Page 5, line 57 |
|  | 6b | Subjects in placebo groups will receive placebo (10 g) dissolved in boiled water, administered orally three times a day for 6 weeks (one sachet per time). They will be allowed to accept standard rehabilitation treatment and use necessary drugs that do not affect the evaluation of study parameters for concomitant diseases. The study drug will be prepared by Shaanxi Buchang Pharmaceuticals Co., Ltd., China. The quality control of the production process strictly adhered to the good manufacturing practice (GMP) of national drug production. | Page 11, line 224 |
| Objectives | 7 | The aims of this basket randomized controlled trial (RCT) are firstly to optimize parameters and examine the feasibility of a subsequent phase III RCT through preliminary evidence on the clinical efficacy and safety of BQTL granules on three diseases (ischemic stroke, stable angina pectoris, and diabetic peripheral neuropathy) involving QDBS syndrome, as compared with placebo. Secondly, it aims to elucidate the mechanism by which BQTL granules exert an effect on QDBS syndrome. | Page 7, line 119 |
| Trial design | 8 | The BOSS trial is designed as a double-blinded, randomized, placebo-controlled, parallel, multicenter, clinical trial. Participants will be randomly assigned to either intervention or control group with an 1:1 allocation using a central randomization system. | Page 4, line 38 |
| **Methods: Participants, interventions, and outcomes** | | | |
| Study setting | 9 | The study is currently open at 14 academic hospitals across China. | Page 8, line 132 |
| Eligibility criteria | 10 | For inclusion, all participants will have a diagnosis of QDBS syndrome and should be aged from 35 years to 80 years. QDBS syndrome follows the Guideline for clinical research of new Chinese medicine.  For the ischemic stroke trial (sub Trial I), patients will be diagnosed with ischemic stroke, with an interval from onset to recruitment of 14‒30 days, and will have a National Institutes of Health Stroke Scale (NIHSS) score ≥ 4 and ≤ 22. Patients will be excluded from sub Trial I if they have a confirmed secondary stroke caused by tumors, brain trauma, or hematological diseases by clinical examination. Patients with other conditions that lead to motor dysfunction (e.g., lameness, osteoarthrosis, rheumatoid arthritis, gouty arthritis), which render a neurological function examination unlikely, will also be excluded.  In the stable angina pectoris trial (sub Trial II), patients will be included if they are diagnosed with stable angina pectoris and have a Canadian Cardiovascular Society classification of Angina Pectoris class I‒III. Patients will be excluded from this trial if they have had acute coronary syndrome or unstable angina pectoris in the previous 3 months, or have other heart diseases (e.g., cardiomyopathy, pericardial disease) such as severe cardiopulmonary insufficiency (congestive heart failure NYHA class IV, severe abnormal pulmonary function), or severe arrhythmias (e.g., rapid atrial fibrillation, atrial flutter, paroxysmal ventricular tachycardia).  In the diabetic peripheral neuropathy trial (sub Trial III) patients with a diagnosis of diabetic peripheral neuropathy will be included. Patients will be excluded if they have HbA1c >10% during the screening period, have had acute, critical diabetes mellitus conditions in the previous 3 months (e.g., hyperglycemia and hypertonic syndrome, diabetic lactic acidosis, diabetic ketoacidosis), or have severe heart disease, brain disease, or kidney disease. Moreover, patients with spinal cord injury, cervical or lumbar vertebral disease (nerve root compression, spinal stenosis, cervical or lumbar vertebra degenerative disease), or sequelae of cerebrovascular disease, neuromuscular junction, or muscular disease; or neuropathies caused by other diseases (e.g., Guillain-Barré syndrome, chronic inflammatory demyelinating polyneuropathy (CIDP), Vitamin B deficiency, hypothyroidism, alcoholism, or severe arteriovenous vasculopathy such as venous embolism, lymphangitis) will be excluded.  Additionally, patients with uncontrolled hypertension (systolic blood pressure ≥ 160 mmHg or diastolic blood pressure ≥ 100 mmHg), or renal or hepatic insufficiency (hepatic insufficiency defined as an alanine aminotransferase (ALT) or aspartate aminotransferase (AST) value that is 1.5 times the upper limit of normal; renal insufficiency defined as a serum creatinine concentration value that is above the upper limit of normal) will be excluded from all trials. Patients with other conditions or mental disorders that, according to the judgment of investigators, would restrict evaluation of mental function or render outcomes or follow-up unlikely to be assessable will also be excluded. Furthermore, pregnant or lactating women, or women who are planning a pregnancy within the next few years, patients who are allergic to the study drug or have a severely allergic constitution, those with a yellow, thick, slimy tongue coating, and those who have participated in other drug or device clinical trials in the past 3 months will also be excluded from all trials. | Page 8, line 143 |
| Interventions | 11a | Eligible patients will be randomized in equal proportions between the BQTL granule groups and placebo groups, receiving either BQTL granules or placebo (10 g) dissolved in boiled water, administered orally three times a day for 6 weeks (one sachet per time). | Page 11, line 224 |
|  | 11b | If the followings occur during the trial, the subjects will withdraw from the trial.  The investigators decided to withdraw:  (1)The subjects are aggravated and unable to continue the trial.  (2)Anaphylaxis or serious adverse events should be stopped according to the doctor's judgment.  (3)If the subjects are found to meet the exclusion criteria after randomization, withdraw the trial medication from the subjects.  (4)Subjects have poor compliance with medication and are not standardized.  (5)Other reasons researchers believe subjects should withdraw from the trial.  The subjects decided to withdraw:  (1)For whatever reason, the subject is unwilling or unlikely to continue the trial, asking the researcher to withdraw from the study and suspending the researcher.  (2)Although the subjects do not explicitly withdraw from the study, they no longer accept medication and testing and are lost to follow-up. |  |
|  | 11c | There are laboratory tests and participants will be required to return the unused tablets and boxes at each follow-up visit. | Page 24, line 514 |
|  | 11d | During the trial, it will be forbidden to use acupuncture, CM decoctions (compound granules), Chinese medicine injections, Chinese patent medicines (including external use), and external washing with traditional Chinese medicine and health products with a composition or efficacy similar to the study drug. Participants will be allowed to accept standard rehabilitation treatment and use necessary drugs that do not affect the evaluation of study parameters for concomitant diseases. The use of drugs should be standardized according to the guidelines, and the name, dosage, times, and time of use must be recorded for analysis and report. | Page 12, line 235 |
| Outcomes | 12 | The primary outcome of the study is improvement in QDBS syndrome, defined as a change in the national approved QDBS scale from before to after the 6-week treatment.  For sub Trial I, secondary outcomes will be the following: Neurological impairment will be evaluated using the NIHSS (Time Frame: baseline, and on days 14, 28, and 42 during treatment). Self-rating symptoms will be evaluated using a visual analog scale (VAS). This will include VAS scores for limb numbness, swelling of hands or feet, and spontaneous sweating (hemilateral sweating) (Time Frame: baseline, and on days 14, 28, and 42 during treatment, as well as at day 14 after treatment). Continuous changes in the Modified Rankin Scale score (Time Frame: baseline, at day 42 during treatment, and at day 90 after onset) will also be recorded. Activities of daily living will be measured using the Barthel Index (BI) score (Time Frame: baseline, at day 42 during treatment, and at day 90 after onset).  For sub Trial II, secondary outcomes will include the following: Changes in the Seattle Angina Questionnaire (SAQ) score (Time Frame: baseline, at day 28 during treatment, at day 14 after treatment, and at day 90 after recruitment). Self-rating symptoms will be evaluated using a VAS for chest tightness, chest pain, palpitation, fatigue, and spontaneous sweating (Time Frame: baseline, and on days 14, 28, and 42 during treatment, as well as at day 14 after treatment).  In sub Trial III, the secondary outcomes will be as follows: Changes in the Toronto Clinical Scoring System (Time Frame: baseline, and on days 14, 28, and 42 during treatment, day 14 after treatment, as well as day 90 after recruitment). Self-rating symptoms will be evaluated using a VAS. Local pain, limb numbness, and paresthesia (e.g., burning sensation, formication, electrical sensation) (Time Frame: baseline, and on days 14, 28, and 42 during treatment, as well as at day 14 after treatment).  For all trials, quality of life will be measured as a secondary outcome, using the 36-Item Short Form Survey (SF-36). (Time Frame: Baseline, at day 42 during treatment, and at day 90 after onset/recruitment).  The safety outcomes will include any adverse events and clinically meaningful changes in vital signs or laboratory parameters during the trial. Participants will be asked to report any abnormal reactions occurring during the trial to the investigators. At week 6, participants will undergo liver function and renal function tests to monitor hepatotoxicity and nephrotoxicity. | Page 13, line 245 |
| Participant timeline | 13 | Please see Table 1. | Page 22, line 504 |
| Sample size | 14 | For a main phase II trial assessing natural drugs, according to the NMPA recommendation (No. 28, 2007 & No. 109, 2018) and data from our pilot study, the sample size should not be less than 60 per treatment arm for each disease to estimate the parameter. We enrolled 432 participants to allow for dropouts. | Page 10, line 187 |
| Recruitment | 15 | Patients who fulfill the screening criteria will be recruited at 14 Good Clinical Practice (GCP)-approved hospitals in China. Local advertisements will be used for recruitment. A contract research organization will monitor the on-schedule recruitment progress and take measures in a timely manner. | Page 10, line 193 |
| **Methods: Assignment of interventions (for controlled trials)** | | | |
| **Allocation:** | | | |
| Sequence generation | 16a | Participants will be randomized into either the BQTL granule group or the placebo group in an 1:1 ratio. A random sequence table will be generated by Strategic Applications Software (SAS, version 9.4, SAS Institute, Inc., Cary, NC, USA). Randomization will be conducted using a central web-based interactive randomization service system (CIMS, Chengdu, China) with permuted blocks. The system automatically randomizes patients and generates the randomization code and drug code corresponding to the assigned treatment. | Page 10, line 202 |
| Allocation concealment mechanism | 16b | Participants will be randomized using central randomization system, which will not release the randomization code until the patient has been recruited into the trial to ensure allocation concealment. | Page 11, line 208 |
| Implementation | 16c | A random sequence table will be generated by Strategic Applications Software. Randomization will be conducted using a central web-based interactive randomization service system, which automatically randomizes patients and generates the randomization code and drug code corresponding to the assigned treatment. | Page 11, line 203 |
| Blinding (masking) | 17a | All the participants, physicians, nurses, data managers, statisticians, and other staff will be blinded to the treatment allocations until the trial is completed.In order to ensure the implementation of the blinding, the pharmaceutical factory specially produces the test drug and placebo with similarities in appearance and flavour. The participants can not judge the drug type according to appearance and flavour. | Page 11, line 210 |
|  | 17b | Two-level unblinding method will be used. When data is all inputted into the database, and after query, verification and blind review, the data will be locked, and the staff who keeps the blind codes will carry out the first-level unblinding. Then the analysts will input the data for statistical analysis. After that, the specific intervention corresponding to each code will be unblinded.  In case of emergency, the principal investigator of each center, who has the only authority to view the blind codes, can log into the central randomization system for emergency unblinding. To maintain the overall quality and legitimacy of the trial, emergency unblinding should occur only in exceptional circumstances when knowledge of the actual treatment is absolutely essential for further management of the subject. Once unblind, the subject with this code should withdraw from the trial as shedding case. | Page 11, line 212 |
| **Methods: Data collection, management, and analysis** | | | |
| Data collection methods | 18a | To promote data quality, each center’s personnel will be trained centrally based on the study requirements, including standardized evaluation of scales involved in the trial, requirements for serum specimen collection, and the eliciting of information from participants in a uniform reproducible manner.  The Evaluation Scale of Qi Deficiency and Blood Stasis Syndrome is a 21-item clinician-rated scale with anchored item descriptions. The scale has been developed, validated, and applied in the Department of Neurology, Cardiology, Endocrinology, and multiple investigators have used this method to assess patients with QDBS syndrome. It has excellent inter-rater reliability and internal consistency. | Page 13, line 248 |
|  | 18b | There will be financial reimbursement, systematic methods and reminders for contacting patients, scheduling appointments, and monitoring retention to improve participant retention. For the participants withdrawing from the study or lost to follow-up, the case data should be retained. The investigators will take active measures to complete the last test as far as possible, and carry forward the last test result to the final result. | Page 15, line 309 |
| Data management | 19 | An electronic data capture system will be used in this study. The investigator/clinical research coordinator (CRC) will input the original data into the electronic data capture system accurately, in a timely, complete, and standard manner. After data entry, data may not be changed at will. If data entries need to be modified, the investigator/CRC will need to record the reason for modification according to the system prompt. All the operations in the system are traceable.  The data manager will develop a detailed data verification plan according to the protocol and case report form, including logic verification, scope verification, time window verification, consistency verification, and compliance verification. The efficiency indicators and key safety indicators should be fully verified to ensure the accuracy and integrity of the data. Data verification should be carried out in the case of an unknown test group, and the generated data query content should avoid deviation or induced questions.  The study documents will be retained in a secure location for at least 5 years after trial completion. | Page 15, line 314 |
| Statistical methods | 20a | Statistical analysis will be performed using Statistical Analysis System version 9.4 (SAS Institute Inc., Cary, North Carolina, USA) statistical software packages. Outcome measurements will be analyzed using full analysis sets and per protocol sets according to intention-to-treat (ITT) analysis. Safety analysis will be performed in a safety set, which is defined as a subset of subjects who received at least one treatment and had actual safety indicators record data. The statistical analysis will include baseline characteristics of participants, compliance and concomitant medication, efficacy analysis and safety analysis. For continuous data, we will describe the results using the mean (standard deviation, SD), maximum, minimum, median and non-parametric test median (quartile deviation, QD). Categorical data will be described as absolute values and proportions. For continuous outcomes, paired t-test or Wilcoxon signed-rank test will be used to analyze significant differences between baseline and each time point. The Wilcoxon rank-sum test will be employed for comparisons between treatment groups. Chi-squared test will be used for categorical data and Wilcoxon rank-sum test will be used for ranked data. Analysis of covariance (ANCOVA) will be used to control potential confounding variables.  Significance tests will be two-tailed, with a statistical probability of P <0.05. Baseline characteristics in each group will be analyzed using descriptive statistics. Compliance analysis will be based on full analysis sets, and analysis of concomitant medications will be based on safety sets. Regarding the primary outcome variable, between-group comparisons of the change in syndrome score will be analyzed between pre- and post-treatment using paired t-test or Wilcoxon signed-rank test. As to the secondary outcomes, comparisons of the changes in NIHSS score in sub Trial I, the Seattle Angina Questionnaire score in sub Trial II, and the Toronto Clinical Scoring System in sub Trial III, will be analyzed using paired t-test or Wilcoxon signed-rank test between baseline and each time point. The Wilcoxon rank-sum test will be employed for comparisons between treatment groups. Any factors impacting efficacy, such as age and sex, should be taken into account as covariate. Safety will be analyzed in terms of all the adverse events occurred during the trial, and the incidence will be compared between groups using the chi-squared test or Fisher’s exact probability method. | Page 16, line 342 |
|  | 20c | The last observation carry forward (LOCF) approach will be used to impute missing data of primary outcome according to intention-to-treat analysis. A sensitivity analysis will be conducted to determine the robustness of the results under the missing at random assumption, to evaluate the role of lost to follow-up (i.e., participants who did not follow-up to V4), by using the LOCF method. And a P value of less than 0.05 will be considered statistically significant. | Page 17, line 370 |
| **Methods: Monitoring** | | | |
| Data monitoring | 21a | The protocol compliance, safety, and the trial data will be supervised by the Data and Safety Monitoring Board (DSMB), which is an independent group of experts that advises funding agencies and study investigators. DSMB members include experts from different fields (Western Medical Sciences, Chinese Medicine, Clinical Epidemiology, and Statistics). | Page 16, line 333 |
| Harms | 22 | All adverse events occurring after entry into the study will be recorded. In case of serious adverse events during the clinical trial, the investigators should immediately take appropriate treatment measures for the subjects, and report to the principal investigator, the ethics committee, the local medical products administration, and the National Medical Products Administration by telephone or fax within 24 hours. Meanwhile, a written report shall be submitted to the above institutions within 15 days. The investigators should follow up the serious adverse events until they are resolved, return to the baseline level, proved unfixable or permanent, switch to other treatment or death. The medical documents related to the serious adverse events should be recorded in the original documents and follow-up documents, including the results of laboratory examinations. | Page 14, line 293 |
| Auditing | 23 | An auditing will be conducted twice a month during the enrollment period and every month during the follow-up period, and the process will be independent from investigators. | Page 16, line 337 |
| **Ethics and dissemination** | | | |
| Research ethics approval | 24 | The trial protocol was approved by the Dongzhimen Hospital, Beijing University of Chinese Medicine (No. DZMEC-JG-2019-161). Patients will receive a detailed information sheet and will complete written consent forms prior to enrollment. | Page 19, line 416 |
| Protocol amendments | 25 | Any modifications to the protocol which may impact on the conduct of the study, patients’ potential benefit or safety will require a formal amendment to the protocol. Such amendment will be agreed upon by sponsor and principal investigator, and approved by the Ethics Committee prior to implementation. Version control will use protocol identifiers and dates, as well as list of amendments, to help track the history of amendments and identify the most recent protocol version. |  |
| Consent or assent | 26a | Trained research clinicians will introduce the trial to patients  who will be shown the consent document regarding the main aspects of the trial. Patients will be able to have an informed discussion with the research clinicians to ask questions. Research clinicians will obtain written consent from patients willing to participate in the trial. | Page 10, line 196 |
|  | 26b | Blood samples of 1/3 subjects will be collected for future exploratory researches.The specimens collection is covered in the informed consent process for the trial. | Page 25, line 524 |
| Confidentiality | 27 | All study-related information will be stored securely at the study site. All participant information will be stored in locked file cabinets in areas with limited access. All specimens, reports, data collection, process, and administrative forms will be identified by a coded number to maintain participant confidentiality. All records that contain names or other personal identifiers, such as locator forms and informed consent forms, will be stored separately from study records identified by code number.Some participants’ tongue and face will be photographed during the research. The photos will be only for scientific research and will cover important parts when published. |  |
| Declaration of interests | 28 | The authors declare no conflict of interest. | Page 19, line 421 |

1. Huang, Z., Liu, F., Sun, Y., Ding, T., Cheng, W., Su Y. Rapid qualitative analysis of chemical constituents of BuqiTongluo Granules by UPLC-Q-TOF-MS^E^ technology combined with UNIFI database[J]. Northwest Pharmaceutical Journal, 2019, 34(04): 477-483. (In Chinese) [↑](#endnote-ref-0)
